# Synthesis of Poly(Lactic Acid-*co*-Glycolic Acid) Copolymers with High Glycolide Ratio by Ring-Opening Polymerisation

**DOI:** 10.3390/polym13152458

**Published:** 2021-07-26

**Authors:** Alastair Little, Alan M. Wemyss, David M. Haddleton, Bowen Tan, Zhaoyang Sun, Yang Ji, Chaoying Wan

**Affiliations:** 1 International Institute for Nanocomposites Manufacturing (IINM), WMG, University of Warwick, Coventry CV4 7AL, UK; alastair.little@warwick.ac.uk (A.L.); A.Wemyss@warwick.ac.uk (A.M.W.); 2 Department of Chemistry, University of Warwick, Coventry CV4 7AL, UK; d.m.haddleton@warwick.ac.uk; 3 PJIM Polymer Scientific Co., Ltd., Shanghai 201102, China; tanbowen@pjchem.com (B.T.); sunzhaoyang@pjchem.com (Z.S.); jiyang@pjchem.com (Y.J.)

**Keywords:** poly(lactic-*co*-glycolic acid), poly(glycolic acid), poly(lactic acid), ring-opening polymerisation, high temperature GPC, biodegradable polymers

## Abstract

The rise in demand for biodegradable plastic packaging with high barrier properties has spurred interest in poly(lactic acid-*co*-glycolic acid) (PLGA) copolymers with a relatively high glycolide content. In this work, we examined how reaction conditions affect the synthesis of PLGA25 (L:G 25:75) through the ring-opening polymerisation of d-l-lactide (L) and glycolide (G), using tin 2-ethylhexanoate (Sn(Oct)_2_) as the catalyst and 1-dodecanol as the initiator. The effects of varying the initiator concentration, catalyst concentration, reaction time, and temperature on the molecular weight, monomer conversion, and thermal properties of PLGA25 were investigated. Increasing the reaction temperature from 130 to 205 °C significantly reduced the time required for high monomer conversions but caused greater polymer discolouration. Whilst increasing the [M]:[C] from 6500:1 to 50,000:1 reduced polymer discolouration, it also resulted in longer reaction times and higher reaction temperatures being required to achieve high conversions. High M_n_ and M_w_ values of 136,000 and 399,000 g mol^−1^ were achieved when polymerisations were performed in the solid state at 150 °C using low initiator concentrations. These copolymers were analysed using high temperature SEC at 80 °C, employing DMSO instead of HFIP as the eluent.

## 1. Introduction

The use of biodegradable plastic packaging is regarded as an effective way to reduce the amount of plastic pollution in the natural environment. After starch blends, poly(lactic acid) (PLA) is the world’s most widely used biodegradable plastic [1]. One factor limiting PLA’s usage is its poor barrier properties in comparison to petroleum-based plastics, such as PET, PE, PP, or EVOH [2,3,4,5,6]. Additionally, PLA requires industrial composting facilities (>58 °C) to undergo biodegradation and is slow to degrade in the ocean and other natural environments [7,8,9]. Therefore, in order to expand the applications of biodegradable plastics, there is a need to develop high performance biodegradable plastics with more controlled hydrolytic degradation over a wider range of conditions [10].

Poly(glycolic acid) (PGA) is a biodegradable aliphatic polyester that has biodegradation rates comparable to cellulose. It possesses a higher tensile strength, superior barrier properties, and higher thermal stability than most currently used packaging plastics [11,12]. As a result of the increased demand for biodegradable packaging, interest in PGA-based packaging has risen due to its exceptional material properties. However, PGA has some limitations, such as its brittleness, high degree of crystallinity (>40%), and high melting temperature (220–230 °C) making it difficult to process. Furthermore, PGA has a high susceptibility towards hydrolytic degradation, which aids biodegradation, but can also result in a short service life when used in packaging due to the concomitant deterioration of its mechanical and barrier properties.

Copolymerisation of glycolide with other monomers can alter the properties of PGA based materials and overcome the above issues. Poly(lactic acid-*co*-glycolic acid) (PLGA) is a common copolymer that has found widespread commercial use in biomedical devices. PLGAs with high glycolide contents possess PGA’s high barrier properties and tensile strength, whilst having lower melting temperatures so they can be processed at lower temperatures [13]. Due to their reduced hydrophobicity and crystallinity in comparison to PLA, PLGAs display much faster biodegradation, whilst maintaining similar mechanical properties to PLA [7]. This has encouraged research into the development of high barrier PLGA based biodegradable packaging. PLGA is currently a relatively high cost material and so suited to high value applications, such as medical devices. Similarly, the price of medical grade PLA is high in comparison to packaging grade PLA [14]. By adapting PLGA production processes towards those used for packaging grade PLA, high barrier biodegradable PLGA packaging materials may be produced. Furthermore, the cost of PLGA may decline as a result of the development of new synthetic routes towards glycolic acid [13,15,16].

High molecular weight PLGA is typically synthesised through the ring-opening polymerisation (ROP) of lactide and glycolide. Whilst a wide variety of organometallic and organic catalysts have proved to be effective in ROP, tin catalysts are preferred in industry due to their low cost and high activity at the elevated temperatures required for melt polymerisation [17,18,19]. For biomedical uses, the residual tin content in the final polymer must be below 20 ppm, meaning that either a low catalyst concentration must be used during synthesis or that excess catalyst is removed from the polymer through a solvent based purification step. Since high glycolide PLGAs are insoluble in common organic solvents, they are typically synthesised using very low catalyst concentrations. Vicryl is an absorbable suture made of PLGA containing 90 mol% of glycolide. Examples in the patent literature state that it is synthesised using low Sn(Oct)_2_ concentrations ([M]:[C] = 50,000:1 to 250,000:1) at high reaction temperatures (≥200 °C) for at least 5 h [20,21]. However, polymer degradation and transesterification under these conditions make it difficult to obtain high molecular weights.

Most commercial PLGA produced for biomedical applications contains at least 50 mol% of lactide. Despite the fact that PLGA is a well-established polymer that has been used for decades in biomedical applications, little has been published examining the synthesis conditions of PLGAs with high glycolide contents. Gilding and Reed published the first major work regarding the ROP of PLGA in 1979, in which some high glycolide PLGAs were synthesised at 200 °C for 4 h [22]. Since then, various authors have examined how the reaction temperature, reaction time, catalyst concentration, and initiator concentration affect the molecular weight (typically inferred from intrinsic viscosity) and conversion of PLGAs with high lactide contents synthesised by ROP [23,24,25,26,27,28]. Many authors have also evaluated the reaction kinetics and the effects of altering reaction parameters on the ROP of PLAs [29,30,31,32].

However, there are wide disparities between the reported synthetic conditions for PLGAs throughout the literature. The faster rate of propagation (*k_p_*) of glycolide relative to lactide and PGA’s higher *T_m_* are contributing factors to this, resulting in the optimum conditions varying depending upon the composition of the PLGA. Therefore, we carried out an extensive study examining how varying the reaction conditions affects the synthesis of PLGAs containing high percentages of glycolide. In order to be commercially viable, high glycolide content PLGAs need to have a high molecular weight, as this increases their thermomechanical properties, hydrolytic stability, and retention of strength during degradation [24,33,34,35]. Due to its low melt viscosity, low molecular weight PLA is only suitable for injection moulding, whereas high molecular weight PLA can be thermoformed, fibre-spun, and film extruded, allowing for a wide variety of additional applications [35,36]. This suggests that in order to be melt-processed using a wide range of techniques, high molecular weight PLGAs would also be required.

In this work, our aim was to synthesise high molecular weight PLGA25 (L:G 25:75) at high conversions (≥96%) and at relatively low reaction temperatures. Therefore, we examined how the initiator concentration, catalyst concentration, reaction temperature, and reaction time affect the molecular weight, monomer conversion, colour, and thermal properties of PLGA25. This L:G composition was selected as it is high in glycolide content whilst still being an amorphous material, which results in transparent products. Once the lactide percentage falls below 15%, the copolymer becomes semicrystalline and has a *T_m_* of about 190 °C [13]. All PLGAs in this study were synthesised by the ROP of glycolide and d-l-lactide using tin 2-ethylhexanoate (Sn(Oct)_2_) as the catalyst and 1-dodecanol as the initiator.

## 2. Materials and Methods

### 2.1. Materials

d-l-lactide was purchased from Arcos Organics (Leicestershire, UK). Glycolide was provided by Pujing Chemicals Ltd. (Shanghai, China) Both were recrystallized from ethyl acetate three times before use. Sn(Oct)_2_ (>96%) and acetone (analytical grade) was purchased from VWR, Alfa Aesar ( Leicestershire, UK). Anhydrous toluene, 1-dodecanol (ACS reagent, ≥98.0%), ethyl acetate (HPLC grade, ≥99.7%), HFIP, DMSO-d6 and DMSO (dry puriss) were purchased from Sigma-Aldrich (Gillingham, UK).

### 2.2. Synthesis of PLGA

For the reactions varying the monomer to 1-dodecanol ([M]:[I]) ratio, 0.015 mol (1.74 g) of glycolide, and 0.005 mol (0.72 g) of d-l-lactide were added to a dried Schlenk flask. This was sealed, attached to a Schlenk line, and purged with N_2_. Solutions containing 1-dodecanol and tin(II) 2-ethylhexanoate in anhydrous toluene were then injected and a vacuum was applied to remove the toluene. The reaction mixture was put under a flow of N_2_, placed in an oil bath at 150 °C and magnetically stirred at 300 rpm for 2.5 h. After 2.5 h, the flask was removed from the oil bath, and the polymer was removed whilst still hot. The polymer was ground using a freezer mill before being analysed.

For the conversion optimisation experiments, a reaction mixture containing 0.04 mol (4.92 g) of monomer and the desired amounts of catalyst and initiator was made. A total of 0.8 g of this mixture was then added to a 15 mL sample vial, sealed, and purged with N2. The vial was then added to an oil bath at a set temperature. After the desired time, the vial was removed, placed in liquid N_2_, and smashed to remove the sample.

### 2.3. Nuclear Magnetic Resonance Spcetroscopy (NMR Spectroscopy)

The 400 MHz ^1^H NMR spectra were recorded at 80 °C on an AV400 Bruker Avance III 400 MHz spectrometer (Coventry, UK) using DMSO-d_6_ as the solvent. For sample preparation, 15 mg of PLGA25 was added to 0.7 mL DMSO-d_6_ and stirred at 80 °C for 30 min.

### 2.4. Size Exclusion Chromatography (SEC)

For SEC analysis performed in DMSO, SEC analysis was carried out on an Agilent PLL220 GPC (Santa Clara, CA, USA) equipped with a differential refractive index detector and a viscometer. DMSO containing 0.1 M of sodium nitrate was used as the eluent at a temperature of 80 °C. Samples were run at 1 mL min^−1^ at 80 °C. Poly(methyl methacrylate) standards (Agilent EasyVials) (Santa Clara, CA, USA) were used for calibration between 2,270,000 and 1860 g mol^−1^ and were fitted with a 3rd order polynomial. Analyte samples were dissolved in DMSO by shaking at 100 °C for roughly 30 min and filtered. Molecular weight and dispersity values of synthesised polymers were determined by conventional calibration using Agilent GPC/SEC software.

For SEC analysis performed in HFIP, HFIP containing 5 mM of sodium trifluoroacetate was used as the eluent at a temperature of 40 °C. The column was a PL-HFIP column. The detector was a differential refractive index (DRI) detector and a poly(methyl methacrylate) calibration was used to determine molecular weight values.

### 2.5. Thermogravimetric Analysis (TGA)

TGA experiments were performed on a Mettler-Toledo TGA (Columbus, OH, USA). Samples were heated under a nitrogen atmosphere over a temperature range of 25–500 °C and at a heating rate of 10 °C min^−1^. Samples of mass 6 mg were used. T_d_ values were evaluated from 5% degradation.

### 2.6. Differential Scanning Calorimetry (DSC)

DSC experiments were performed on a Mettler-Toledo DSC (Columbus, OH, USA). Samples were scanned under a nitrogen atmosphere over a temperature range of 25–230 °C and at a heating rate of 10 °C min^−1^. Samples of a mass of 6 mg were used. *T_g_* values were reported from the second heating scans.

## 3. Results and Discussion

### 3.1. Initiator Concentration

The ROP of cyclic esters is often initiated by hydroxyl compounds, where a reduction in the concentration of the initiator relative to the monomer increases the molecular weight of the product. 1-Dodecanol is frequently used as an initiator in the ROP of PLGA, as its high boiling point prevents evaporation during polymerisation and its relatively high Log*P* value reduces the ingress of water into the reaction. When synthesising PLGA75, Wang et al. varied the monomer to the 1-dodecanol ratio ([M]:[I]) from 20:1 to 300:1 and observed that the M_n_ increased from 3400 to 99,000 g mol^−1^ [24]. Elsewhere in the literature, researchers have used even lower 1-dodecanol concentrations to target higher molecular weights (>100,000 g mol^−1^) for PGA, PLA, and PLGA [16,23,27,28,29,37].

When performing the ROP of lactide and glycolide in bulk at high temperatures, the obtained molecular weights are not always reflective of the [M]:[I] due to side reactions, such as transesterification, occurring alongside chain propagation. Therefore, in order to examine the relationship between the initiator concentration and molecular weight in PLGA25, a series of PLGA25s were synthesised in which the [M]:[I] was varied from 30:1 to 30,000:1. These experiments were all performed at 150 °C over 2.5 h using a monomer to catalyst ratio ([M]:[C]) of 6500:1.

Due to their poor solubility in common solvents, the molecular weight of high glycolide PLGAs are typically determined by SEC using hexafluoroisopropanol (HFIP) as the eluent. However, as HFIP is expensive and quite hazardous we used DMSO at 80 °C as a low-cost and safer alternative. As the only reported use of SEC in DMSO at high temperatures for similar polymers has been for PGA oligomers, PLGA25 synthesised using an [M]:[I] = 30:1 was also analysed with SEC in HFIP at 40 °C and the results from the two eluents were compared (Table S1) [38]. Both SEC systems gave similar results, in DMSO at 80 °C, M_n_ = 23,400 g mol^−1^ (*Ð* = 1.56) was recorded and in HFIP at 40 °C, M_n_ = 25,000 (*Ð* = 1.93) was recorded. Thus, DMSO at 80 °C is an excellent alternative eluent to HFIP for conducting SEC experiments with high glycolide PLGAs.

**Figure 1:**
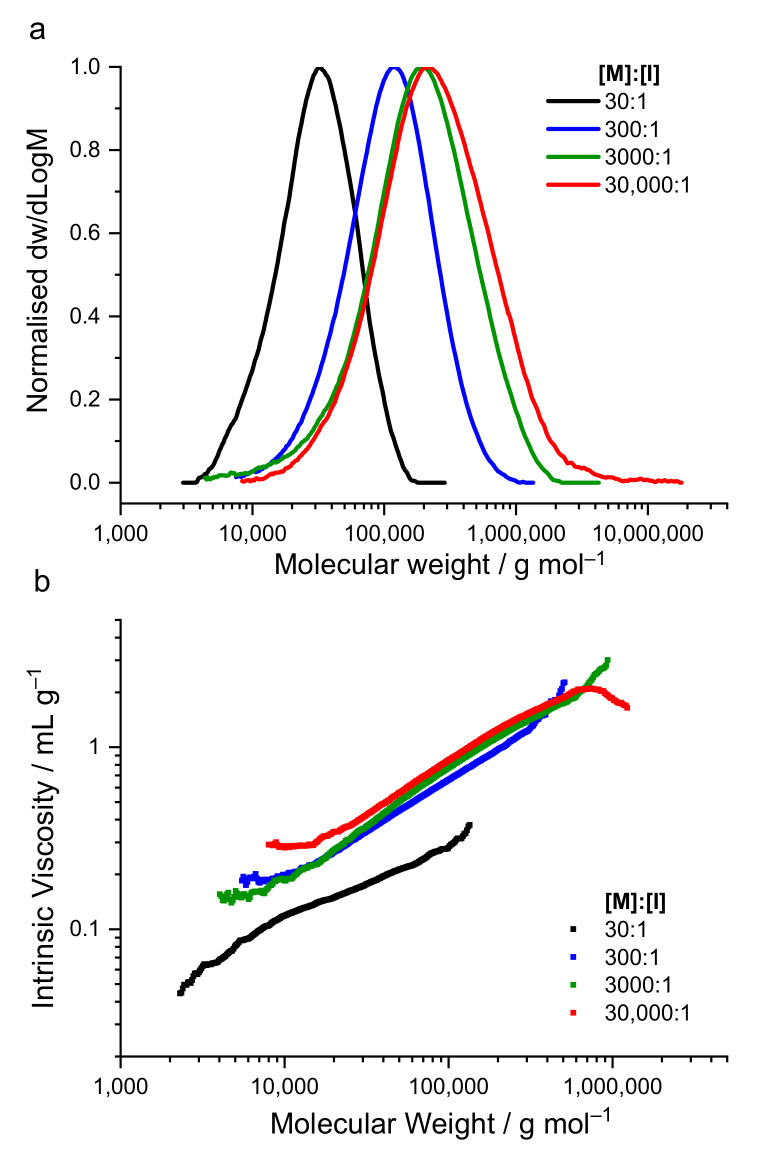
(**a**) SEC traces and (**b**) Mark–Houwink–Sakurada (MHS) plots for PLGA25 synthesised using various [M]:[I] ratios. SEC analysis was performed in DMSO at 80 °C using a PMMA calibration. All polymers were synthesised at 150 °C for 2.5 h under N_2_, using an [M]:[C] = 6500:1.

From the SEC analysis shown in Figure 1a and Table 1, it can be observed that the molecular weight of PLGA25 increased with decreasing initiator concentration. Using an [M]:[I] = 30,000:1, very high M_n_ and M_w_ values of 136,000 g mol^−1^ and 399,000 g mol^−1^ were achieved. The increase in molecular weight was accompanied by an increase in dispersity from 1.56 to 2.92, suggesting that more side reactions, such as intermolecular transesterification, occurred as higher molecular weight polymers were formed.

Unexpectedly, greater discolouration occurred as the 1-dodecanol concentration was reduced (Figure S2). The PLGA25 turned from white to orange/brown as the M_n_ increased from 23,400 to 136,000 g mol^−1^. Discolouration is a common problem encountered in the synthesis of polyesters by both polycondensation and ROP and has been reported to be influenced by the catalyst used [39,40]. During PLA synthesis, discolouration is frequently observed at higher reaction temperatures (>180 °C) and longer reaction times and is generally attributed to side reactions and polymer degradation [41]. However, these experiments were performed at relatively low temperatures (150 °C) and the only variable changed was the 1-dodecanol concentration, which led to an increase in molecular weight and discolouration.

Mark–Houwink–Sakurada (MHS) measurements were also obtained to observe whether any changes in structure occurred with the change in molecular weight. The MHS plots for the three high molecular weight PLGA25s synthesised using [M]:[I] = 3000:1–30,000:1show good linearity over a wide molecular weight range with α values of 0.55–0.59, suggesting linear polymers (Figure 1b and Table S2). The low molecular weight PLGA25 synthesised using an [M]:[I] = 30:1 had a much lower intrinsic viscosity and α value = 0.37. This lower intrinsic viscosity suggests this sample contains some cyclic polymers formed from back-biting reactions. Previous researchers have shown cyclic PLA’s to have lower intrinsic viscosities than their linear counterparts [41,42].

The ^1^H NMR spectra revealed that as the [M]:[I] increased, the total monomer conversion (C_LD+GD_) decreased from 98.8 to 94.0%. For each polymerisation, the glycolide conversion (C_GD_) was high, however, the lactide conversion (C_LD_) decreased from 98.0% to 76.9%, indicating that the decrease in total monomer conversion was due to a decrease in the lactide conversion. It is known that glycolide has a faster rate of propagation than lactide often resulting in PLGA having a lower L:G ratio than the monomer feed [22]. This drop in lactide conversion resulted in a lower lactide content in the final PLGA. It has been reported that increasing the 1-dodecanol concentration increases the reaction rate, leading to shorter reaction times being required to achieve high conversions [18,29,32]. Therefore, longer reaction times are needed to achieve higher lactide conversions at lower 1-dodecanol concentrations.

### 3.2. Reaction Temperature, Time, and Catalyst Concentration

As the lactide conversion for the high molecular weight PLGA25 synthesised using an [M]:[I] = 30,000:1 was relatively low, further reaction optimisation was required. Aside from causing a poor yield, a low conversion is undesirable as it results in higher traces of a residual monomer in the polymer product. Residual lactide and glycolide increase the rate of hydrolytic degradation and result in poorer mechanical properties, decreasing the product life-time. Additionally, they cause degradation during melt-processing [35,43]. Therefore, they require removal after synthesis, increasing production costs. Reaction conversions above 96% are generally considered high in polylactides synthesised by ROP [22,44].

**Figure 2:**
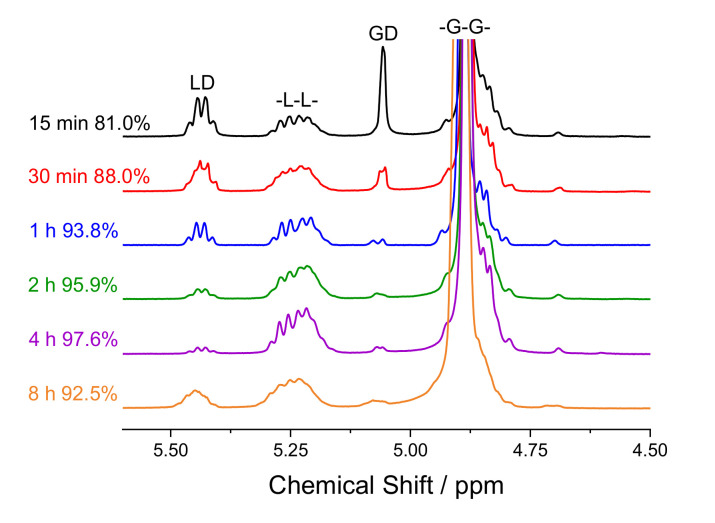
^1^H NMR spectra of PLGA25 synthesised at 150 °C at 15 min to 8 h using an [M]:[C] = 6500:1 and [M]:[I] = 30,000:1. Conversion values are for the total monomer conversion. LD = lactide, -L-L- = PLA, GD = glycolide, and -G-G- = PGA.

A series of experiments were performed to examine the effects of reaction time and temperature on monomer conversion. To begin, PLGA25 was polymerised at 150 °C using an [M]:[C] = 6500:1 and [M]:[I] = 30,000:1 at times ranging from 15 min to 8 h. The ^1^H NMR spectra of these polymers revealed how conversion changed with time, Figure 2. Conversion initially increased, reaching a peak (C_LD+GD_ = 97.6%) at 4 h, and then fell to 92.5% after 8 h due to thermal depolymerisation. Similarly, both M_n_ and M_w_ initially increased, but then started to decrease after 1 h, most likely due to random chain scission during the reaction. However, the conversion improved significantly during this time, increasing from 93.8% to 97.6% when the reaction time was increased from 1 to 4 h. Similar observations of reductions in molecular weight and conversion after certain periods of time have been reported previously for PLA and PLGA synthesised using Sn(Oct)_2_ [23,29,30,31].

PLGA25 is an amorphous polymer that melts at around 180–190 °C, thus at 150 °C the reaction occurs in the solid-state. Performing PLA and PGA polymerisation at low temperatures in the solid state has been shown to be an effective way to reduce the remaining monomer content and achieve high conversions [11,31,45,46]. However, in order to see whether the conversion could be increased further, the reaction temperature was increased to 180 °C and subsequently to 205 °C, in order to perform reactions in the melt state. Total monomer conversions of 98.0% and 98.1% were achieved in a 2 h reaction at 180 °C and a 2 h reaction started at 180 °C and increased to 205 °C after 30 min, respectively. However, the PLGA25 formed at these temperatures showed much greater discolouration (Figure S3). The PLGA25 formed at 180–205 °C was a very dark brown polymer with a low M_n_ (45,900 g mol^−1^), suggesting higher amounts of decomposition. Since these higher reaction temperatures increased PLGA25 discolouration, some polymerisation reactions were then performed at 130 °C. The resulting PLGA25 was much lighter in colour, however a longer reaction time (8 h) was required to achieve a 96.9% total monomer conversion.

Table 2 and Figure S5 report the full conversion and molecular weight data for the synthesis of PLGA25 under these reaction conditions. In general, as the reaction temperature increased from 130 to 150 to 180 °C, the time required to achieve a high total monomer conversion (>96%) fell from 8 to 4 to 2 h, respectively, with similar M_n_ (79,600–94,300 g mol^−1^) and M_w_ (176,000–191,000 g mol^−1^) values achieved in each reaction. However, this increase in reaction temperature was also accompanied by increased discolouration, with the PLGA25 turning from beige to orange to dark brown as the temperature increased from 130 to 150 to 180 °C, suggesting increased polymer degradation (Figure S3). These samples were all of similar molecular weight, so here discolouration was caused by the increase in reaction temperature.

For the ROP of lactide and glycolide, the optimum reaction time and temperature are strongly affected by the catalyst concentration [23,29,41]. Lowering the catalyst concentration leads to higher polymerisation temperatures or longer reaction times being required to reach high monomer conversions [23,47]. However, a lower Sn(Oct)_2_ concentration can improve the polymer’s thermal and hydrolytic stability [47,48,49]. Given the concerns surrounding the toxicity of tin catalysts, a lower catalyst concentration is desirable so to reduce the catalyst residues that will be released into the compost upon biodegradation. As the Food and Drug Administration (FDA) has set an upper limit for the residual tin content in bioabsorbable polymers of 20 ppm, most industrial processes for the synthesis of high glycolide PLGAs employ Sn(Oct)_2_ concentrations below 20 ppm and consequently use long reaction times or high reaction temperatures [20,21,50]. Many researchers have examined the use of very low tin catalyst concentrations in the synthesis of biodegradable biomedical polymers, such as poly(ε-caprolactone) [51,52]. Therefore, a selection of the above experiments was repeated using an [M]:[C] = 50,000:1, which is equal to 20 ppm of Sn(Oct)_2_.

When reducing the catalyst concentration, higher temperatures and longer reaction times were needed to achieve high (>96%) conversion values, Table 2 and Figure S6. At 150 °C, 16 h were required to achieve a high total monomer conversion (97.4%), however this prolonged reaction time resulted in a lower molecular weight as a result of depolymerisation. In order to decrease the reaction time, the reaction temperature was increased to 170 °C. An 8 h reaction at 170 °C yielded a conversion of 96.4%. Further increasing the reaction temperature to 180–205 °C produced a high conversion of 95.9% after 2 h, but resulted in a low M_n_ (59,000 g mol^−1^) and significant discolouration (Figure S4), as was observed at the same temperature when a higher concentration of Sn(Oct)_2_ was used. Similar relationships between the Sn(Oct)_2_ concentration, conversion, and molecular weight have been previously reported by Avgoustakis et al., who when synthesising PLGA70 (L:G = 70:30) achieved the highest conversions and intrinsic viscosities after 4 h reactions at 190 °C using 0.03 wt.% Sn(Oct)_2_ and at 130 °C using 0.1 wt.% Sn(Oct)_2_ [23].

### 3.3. Thermal Properties

DSC and TGA analysis were then carried out for a selection of PLGA25 copolymers, revealing the effects of the M_n_ on their thermal properties (Table 3, Figures S7 and S8). Thermal analysis was performed after the PLGA25s were purified by washing the ground polymer in acetone, followed by removal of the volatiles. DSC analysis showed that as the M_n_ increased from 23,400 to 74,300 g mol^−1^, the *T_g_* increased from 31.1 to 39.3 °C. As the M_n_ increased to 111,000 and 136,000 g mol^−1^, the *T_g_* increased much more slowly. TGA analysis showed that the *T_d_* (5%) increased from 238 to 267 °C as the M_n_ increased from 23,400 to 136,000 g mol^−1^. This indicates that the high molecular weight PLGA25 has a higher thermal stability and would be more stable during melt processing.

The effect of catalyst concentration on the thermal stability of PLGA25 was then examined by analysing two PLGA25 samples synthesised using different Sn(Oct)_2_ concentrations. Both polymers had similar molecular weights, the same L:G ratio, and were synthesised at 150 °C. TGA was performed on the crude polymer samples and showed that increasing the [M]:[C] ratio from 6500:1 to 50,000:1 improved the *T_d_* from 262 to 280 °C (Figure S9). This result highlights that larger amounts of residual Sn(Oct)_2_ in the polymer reduces the thermal stability, as Sn(Oct)_2_ catalyses depolymerisation reactions.

## 4. Conclusions

When synthesising PLGA25 by ROP at 150 °C, increasing the ratio of the monomer to 1-dodecanol ([M]:[I]) from 30:1 to 30,000:1 increased the M_n_ from 23,400 to 136,000 g mol^−1^. This increase in M_n_ was accompanied by increases in dispersity and discolouration. When using a monomer to catalyst ratio ([M]:[C]) of 6500:1, increasing the reaction temperature reduced the time required to achieve high monomer conversions (>96%), such that reactions for 8 h at 130 °C, 4 h at 150 °C and 2 h at 180 °C yielded similar conversion and M_n_ values. However, increasing the reaction temperature resulted in greater polymer discolouration, causing the PLGA25 to turn from beige to dark brown. Increasing the [M]:[C] from 6500:1 to 50,000:1 decreased polymer discolouration, but resulted in longer reaction times (8–16 h) and higher temperatures (150–170 °C) being required to achieve high conversions. Thermal analysis showed that increasing the molecular weight increased the *T_g_* from 31.1 to 41.6 °C and improved the *T_d_* (5%) from 238 to 267 °C. An increase in the thermal stability was also found upon reducing the catalyst concentration. These results demonstrate how reaction variables can be altered in order to achieve high molecular weights and conversions in the synthesis of high glycolide PLGAs for packaging applications.

## Figures and Tables

**Table 1:** Conversion, molecular weights, and thermal properties of PLGA25 synthesised using [M]:[I] = 30:1 to 30,000:1. All polymers were synthesized using an L:G feed ratio of 25:75. C_LD_ = lactide conversion, C_GD_ = glycolide conversion, and C_LD+GD_ = total monomer conversion. All polymers were synthesised at 150 °C for 2.5 h under N_2_, using an [M]:[C] = 6500:1.

[M]:[I]	L:G ^a^	C_LD_^a^/%	C_GD_^a^/%	C_LD+GD_^a^/%	M_n_^b^/g mol^−1^	*Ð* ^b^
30:1	26:74	97.6	99.1	98.7	23,400	1.56
300:1	25:75	97.1	99.2	98.6	74,300	1.92
3000:1	22:78	84.7	98.9	95.3	111,000	2.47
30,000:1	20:80	76.9	99.0	93.7	136,000	2.92

^a^ Determined by ^1^H NMR spectroscopy in DMSO-*d_6_* at 80 °C. ^b^ Determined by SEC in DMSO at 80 °C calibrated using PMMA standards.

**Table 2:** Conversion and molecular weight data for the synthesis of PLGA25 using different [M]:[C] ratios and [M]:[I] = 30,000:1. C_LD+GD_ = total monomer conversion.

[M]:[C] = 6500:1					[M]:[C] = 50,000:1				
Temp/°C	Time/h	C_LD+GD_^a^/%	M_n_^b^/g mol^−1^	*Đ* ^b^	Temp/°C	Time/h	C_LD+GD_^a^/%	M_n_^b^/g mol^−1^	*Đ* ^b^
130	2	92.4	81,600	2.12	150	2	79.5	75,700	1.88
/	4	94.5	64,300	2.07	/	4	90.5	91,300	2.27
/	8	96.9	86,700	2.08	/	8	93.6	117,000	1.82
/	/	/	/	/	/	16	97.4	55,300	1.79
150	0.25	81.0	105,000	2.72	150–170	2, 4	94.9	64,100	2.70
/	0.5	88.0	109,000	2.33	/	/	/	/	/
/	1	93.8	134,000	2.19	170	2	88.9	140,000	2.12
/	2	95.9	79,300	2.08	/	4	94.3	59,300	2.06
/	4	97.6	79,600	2.21	/	6	95.7	89,500	2.60
/	8	92.5	67,200	2.33	/	8	96.4	77,800	3.12
/	/	/	/	/	/	/	/	/	/
180	1	97.2	92,800	1.78	180	1	81.3	112,000	1.99
/	2	98.0	96,000	1.99	/	2	92.2	72,000	2.81
/	/	/	/	/	/	/	/	/	/
180–205	0.5, 1.5	98.1	45,900	2.26	180–205	0.5, 1.5	95.9	59,000	2.62

^a^ Determined by ^1^H NMR Spectroscopy in DMSO-*d_6_* at 80 °C. ^b^ Determined by SEC in DMSO at 80 °C calibrated using PMMA standards.

**Table 3:** Thermal properties of PLGA25 of varying molecular weights. *T_g_* = glass transition temperature. *T_d_* = temperature of 5% degradation.

L:G ^a^	M_n_^b^/g mol^−1^	*Ð* ^b^	*T_g_*^c^/°C	*T_d_^d^*/°C
26:74	23,400	1.56	31.1	238
25:75	74,300	1.92	39.3	255
22:78	111,000	2.47	39.9	261
20:80	136,000	2.92	41.6	267

^a^ Determined by ^1^H NMR spectroscopy in DMSO-*d_6_* at 80 °C. ^b^ Determined by SEC in DMSO at 80 °C calibrated using PMMA standards. ^c^ Determined from DSC. ^d^ Determined from TGA. All polymers were synthesised at 150 °C for 2.5 h under N_2_, using an [M]:[C] = 6500:1.

## Data Availability

The raw/processed data required to reproduce these findings cannot be shared at this time as the data also forms part of an ongoing study.

## Supplementary Materials

The following are available online at https://www.mdpi.com/article/10.3390/polym13152458/s1, Figure S1: ^1^H NMR spectrum of PLGA25 synthesised at 150 °C for 2.5 h using an [M]:[C] = 6500:1 and [M]:[I] = 30:1. Spectrum run in DMSO-*d_6_* at 80 °C and 400 MHz. Table S1: Molecular Weight of PLGA25 synthesised at 150 °C for 2.5 h using an [M]:[C] = 6500:1 and [M]:[I] = 30:1. SEC in DMSO at 80 °C and SEC in HFIP at 40 °C. Table S2: α and K values calculated from Mark–Houwink–Sakurada (MHS) plots of PLGA25s synthesised at 150 °C for 2.5 h using varying [M]:[I] ratios (30:1 to 30,000:1) and [M]:[C] = 6500:1. Determined via SEC in DMSO at 80 °C. Figure S2: Appearances of PLGA25s synthesised at 150 °C for 2.5 h using [M]:[C] of 6500:1 and [M]:[I] of 30:1, 300:1 and 3000:1. Figure S3: Appearances of PLGA25s synthesised using an [M]:[C] = 6500:1 and [M]:[I] = 30,000:1 at 130–205 °C. Figure S4: Appearances of PLGA25s synthesised using an [M]:[C] = 50,000:1 and [M]:[I] = 30,000:1 at 150–205 °C. Figure S5: Graphs of (a) Conversion vs. Time and (b) M_n_ vs. Time for PLGA25 polymerisation reactions performed using an [M]:[C] = 6500:1 and [M]:[I] = 30,000:1 at 130 °C, 150 °C, 180°C and 180–205 °C. Figure S6: Graphs of (a) Conversion vs. Time and (b) M_n_ vs. Time for PLGA25 polymerisation reactions performed using an [M]:[C] = 50,000:1 and [M]:[I] = 30,000:1 at 150 °C, 150–170 °C, 170 °C, 180 °C and 180–205 °C. Figure S7: DSC thermograms of PLGA25 synthesised at 150 °C for 2.5 h using an [M]:[C] = 6500:1 and [M]:[I] = 30:1 to 30,000:1. Figure S8: TGA thermograms of PLGA25 synthesised at 150 °C for 2.5 h using an [M]:[C] = 6500:1 and [M]:[I] = 30:1 to 30,000:1. Figure S9: TGA thermograms of PLGA25 synthesised using different catalyst concentrations. [M]:[C] = 6500:1 and 50,000:1. Both samples were synthesised at 150 °C using an [M]:[I] = 30,000:1.
