# Supplementary material for: Synthesis of Poly(Lactic Acid-co-Glycolic Acid) Copolymers with High Glycolide Ratio by Ring-Opening Polymerisation
Source: Polymers (Basel). 2021 Jul 26;13(15):2458. doi: 10.3390/polym13152458 (PMC8348705; doi:10.3390/polym13152458)
Supplement: Supplementary file 1 [file polymers-13-02458-s001.zip › polymers-1286923-SI.pdf]

Supplementary Information

Supporting Figures and Tables

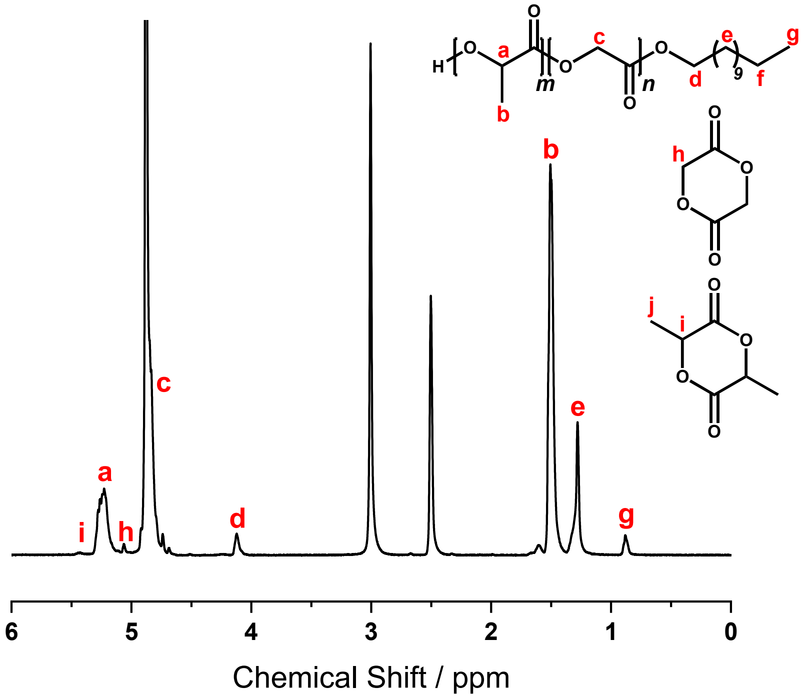

Figure S1. ^{1}H NMR spectrum of PLGA25 synthesised at 150 °C for 2.5 hours using an [M]:[C] = 6,500:1 and [M]:[I] = 30:1. Spectrum run in DMSO-d_6 at 80 °C and 400 MHz.

Table S1. Molecular Weight of PLGA25 synthesised at 150 °C for 2.5 hours using an [M]:[C] = 6,500:1 and [M]:[I] = 30:1. SEC in DMSO at 80 °C and SEC in HFIP at 40 °C.

M]:[I]	M_n[a] / g mol^{-1}	Đ[a]	M_n[b] / g mol^{-1}	Đ[b]
30:1	23,400	1.56	25,000	1.93

[a] Determined by SEC in DMSO at 80 °C calibrated using PMMA standards. [b] Determined by SEC in HFIP at 40 °C calibrated using PMMA standards.

Table S2. α and K values calculated from Mark–Houwink–Sakurada (MHS) plots of PLGA25s synthesised at 150 °C for 2.5 hours using varying [M]:[I] ratios (30:1 to 30,000:1) and [M]:[C] = 6,500:1. Determined via SEC in DMSO at 80 °C.

[M]:[I]	α	K × 10^5 / dL g^{-1}
30:1	0.37	376
300:1	0.57	101
3,000:1	0.59	82
30,000:1	0.55	159

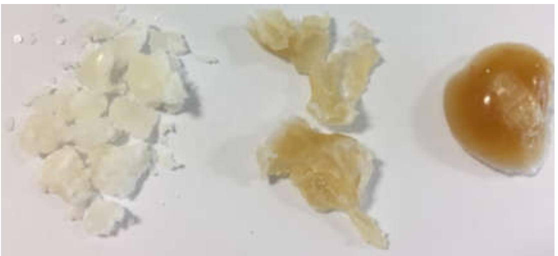

Figure S2. Appearances of PLGA25s synthesised at 150 °C for 2.5 hours using [M]:[C] of 6,500:1 and [M]:[I] of 30:1, 300:1 and 3,000:1.

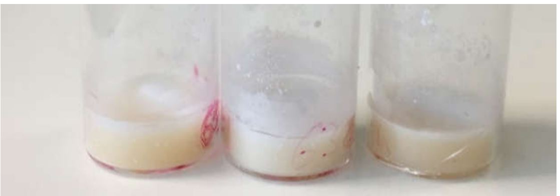

130 °C, 2 h, 4 h, 8 h

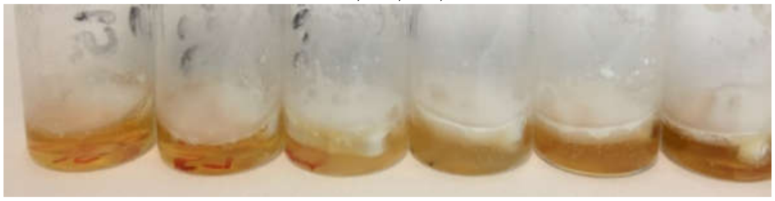

150 °C, 0.15 h, 0.3 h, 1 h, 2 h, 4 h, 8 h

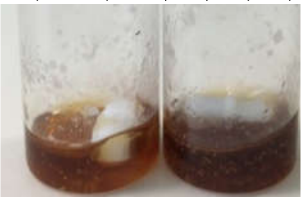

180 °C, 1 h, 2 h

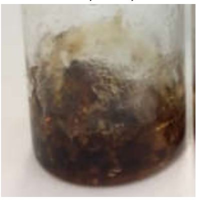

180-205 °C, 2 h

Figure S3. Appearances of PLGA25s synthesised using an [M]:[C] = 6,500:1 and [M]:[I] = 30,000:1 at 130-205 °C.

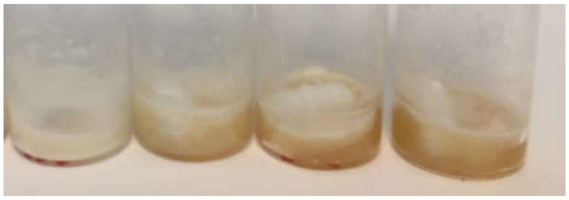

150 °C, 2 h, 4 h, 8 h, 16 h

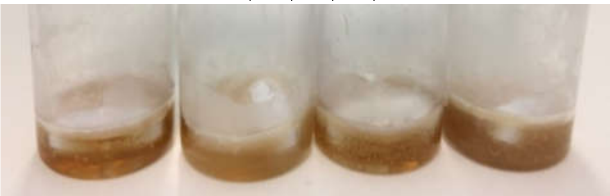

170 °C, 2 h, 4 h, 6 h, 8 h

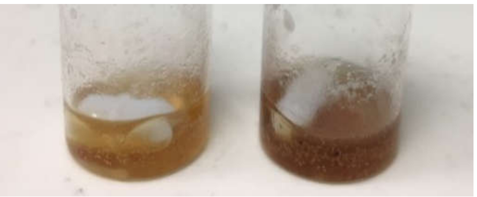

180 °C, 1 h, 2 h

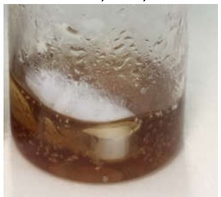

180-205 °C, 2 h

Figure S4. Appearances of PLGA25s synthesised using an [M]:[C] = 50,000:1 and [M]:[I] = 30,000:1 at 150-205 °C.

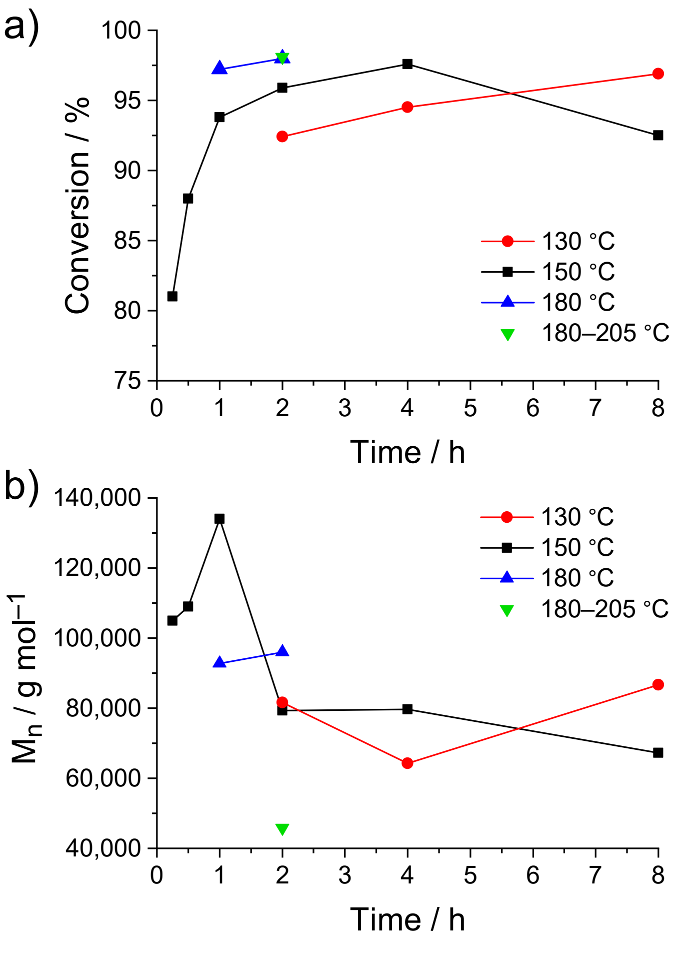

Figure S5. Graphs of a) Conversion vs Time and b) M_n vs Time for PLGA25 polymerisation reactions performed using an [M]:[C] = 6,500:1 and [M]:[I] = 30,000:1 at 130 °C, 150 °C, 180 °C and 180-205 °C.

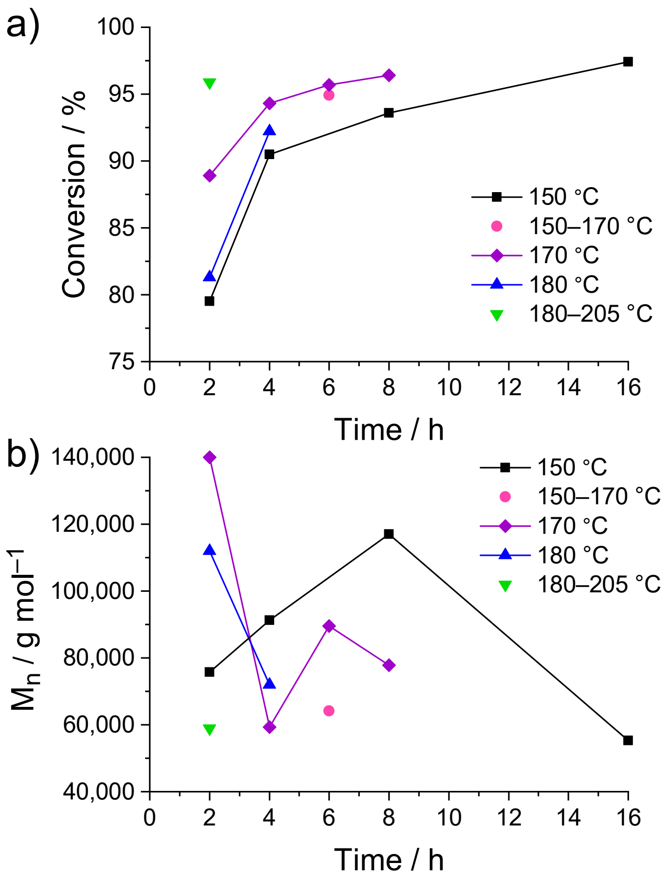

Figure S6. Graphs of a) Conversion vs Time and b) M_n vs Time for PLGA25 polymerisation reactions performed using an [M]:[C] = 50,000:1 and [M]:[I] = 30,000:1 at 150 °C, 150-170 °C, 170 °C, 180 °C and 180-205 °C.

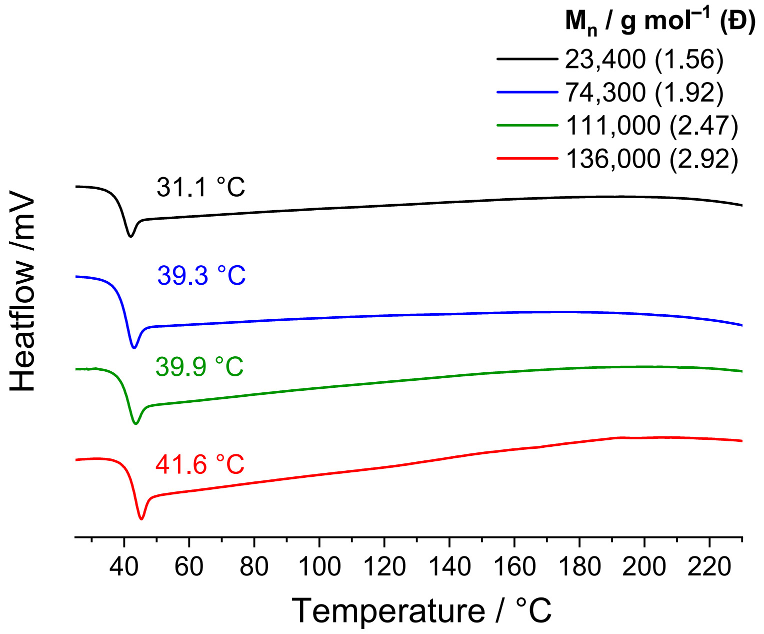

Figure S7. DSC thermograms of PLGA25 synthesised at 150 °C for 2.5 hours using an [M]:[C] = 6,500:1 and [M]:[I] = 30:1 to 30,000:1.

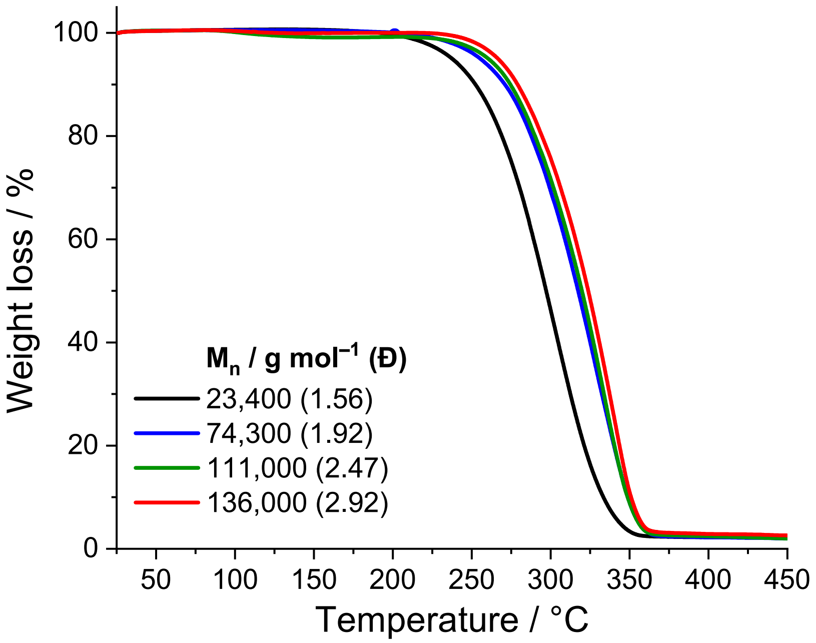

Figure S8. TGA thermograms of PLGA25 synthesised at 150 °C for 2.5 hours using an [M]:[C] = 6,500:1 and [M]:[I] = 30:1 to 30,000:1.

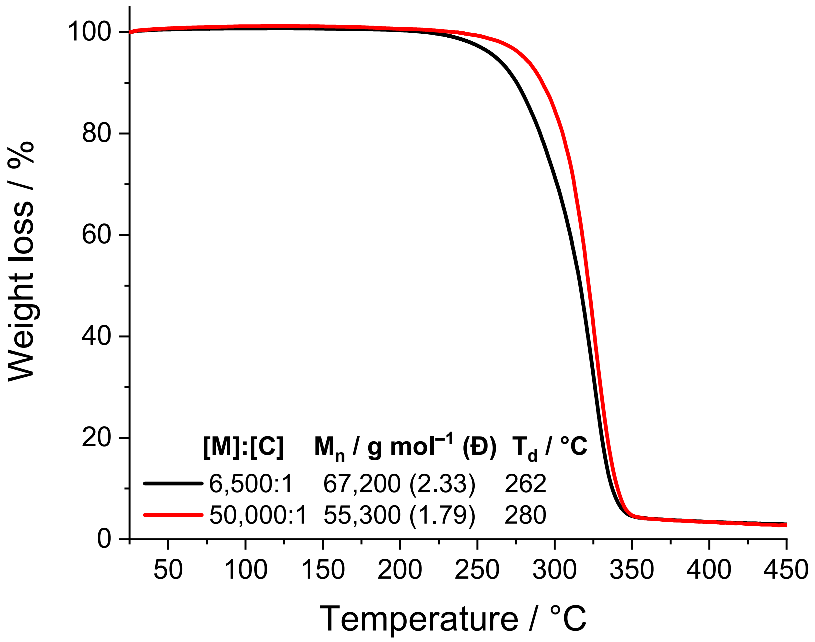

Figure S9. TGA thermograms of PLGA25 synthesised using different catalyst concentrations. [M]:[C] = 6,500:1 and 50,000:1. Both samples were synthesised at 150 °C using an [M]:[I] = 30,000:1.
